# Hepatitis E Infection in HIV-Infected Patients

**DOI:** 10.3389/fmicb.2019.01425

**Published:** 2019-06-26

**Authors:** Antonio Rivero-Juarez, Pedro Lopez-Lopez, Mario Frias, Antonio Rivero

**Affiliations:** Infectious Diseases Unit, Instituto Maimonides de Investigación Biomédica de Córdoba (IMIBIC), Hospital Universitario Reina Sofía de Córdoba, Universidad de Córdoba, Córdoba, Spain

**Keywords:** HIV, HEV, epidemiology, zoonoses, treatment, prevention, diagnosis

## Abstract

**Background:**

The hepatitis E virus (HEV) represents a major cause of acute hepatitis worldwide. The majority of HEV cases occur in low-income countries, mainly Asia and Africa, where HEV causes large outbreaks associated with the consumption of contaminated water and high mortality in specific populations. In high-income countries, HEV infection is considered a zoonotic disease that is linked to the consumption of contaminated food. Although a high proportion of cases have self-limiting asymptomatic or subclinical infections, immunosuppression may modify the pathogenesis and clinical impact of this emerging disease.

**Results and Discussion:**

Here, we review the current knowledge about the epidemiology, diagnosis, clinical manifestations, management and prevention of HEV infection in HIV-infected subjects.

**Conclusions:**

Despite the increasing knowledge about the pathogenesis, epidemiology and clinical impact of HEV infection, several major factors are faced by HIV-infected patients, including treatment recommendations, immunization and risk practices.

## Introduction

The World Health Organization (WHO) estimates that, every year, more than 20 million people worldwide become infected with the hepatitis E virus (HEV) (World Health Organization [Who], 2017). Furthermore, it is estimated that 3.3 million of these cases are symptomatic and produce 44,000 deaths (World Health Organization [Who], 2017). Considering these statistics, the WHO ranks HEV as the leading cause of global acute hepatitis of viral origin. The majority of cases are reported in low-income countries, mainly Asia and Africa, where HEV causes large outbreaks associated with the consumption of contaminated water and is producing by HEV genotypes 1 and 2 (World Health Organization [Who], 2014). In contrast, in high-income countries, the majority of cases are produced by HEV genotype 3 and are linked to the consumption of contaminated food, including mainly pork-derived products and game meat (Faber et al., 2018). Because of the efficient transmission of the infection by this route, the European Food Safety Authority (EFSA) has indicated that HEV infection is a major public health problem in Europe (EFSA, 2017). Although a high proportion of these infections cause self-limiting asymptomatic or subclinical hepatitis (Kamar et al., 2012), there are clinical situations that can produce a worsened prognosis of the infection (Kamar et al., 2012; McPherson et al., 2018), and can even present with acute extrahepatic manifestations (Pischke et al., 2017); overall alterations in the central and peripheral nervous systems (Dalton et al., 2016).

HIV-infected patients encompass immunological, epidemiological, and clinical characteristics that can modify the pathogenesis of HEV. In this sense, after an acute HEV infection, the virus can persist and can develop into a chronic infection (Kenfak-Foguena et al., 2011). Furthermore, the immunosuppression derived from HIV infection can modify the immune response to HEV infection, causing a serological and virological pattern that implies a modification in the diagnosis algorithm (Pineda et al., 2014; Kuniholm et al., 2016). Additionally, in these patients, HEV reinfection has been suggested (Rivero-Juarez et al., 2017b); thus, extra preventive measures should be recommended in these subjects, even in those with evidence of past HEV infection. Furthermore, in patients with underlying chronic liver diseases (mainly by coinfection with other hepatotropic viruses such as hepatitis C or B), acute HEV infection has a worsened prognosis that is associated with a high mortality rate (Péron et al., 2006; Dalton et al., 2008). Thus, due to the high prevalence of hepatitis C and B coinfection among HIV infected patient, the risk of liver decompensation could be high in this population. Finally, HIV-infected subjects may be at higher risk for HEV acquisition due to HIV infection *per se* or to associated risk practices (Payne et al., 2013; Riveiro-Barciela et al., 2014). Thus, HIV-infected patients represent a population that is highly sensitive to HEV infection. For this reason, in this review, we describe the current knowledge about the epidemiology, diagnosis, clinical manifestations, management and prevention of HEV infection in HIV-infected populations.

## Results and Discussion

### Search Strategy and Selection Criteria

The references used in this review were identified through searches of the PubMed database with the search terms “Hepatitis E,” “chronic Hepatitis E,” “HIV,” and “viral hepatitis” from 1990 until September 2018. Articles were also identified through searches of the authors’ own files. Only papers published in English were reviewed. The final reference list was generated on the basis of originality and relevance to the broad scope of this review.

### Epidemiology of HEV in HIV-Infected Patients

#### Prevalence and Incidence

**FIGURE 1:**
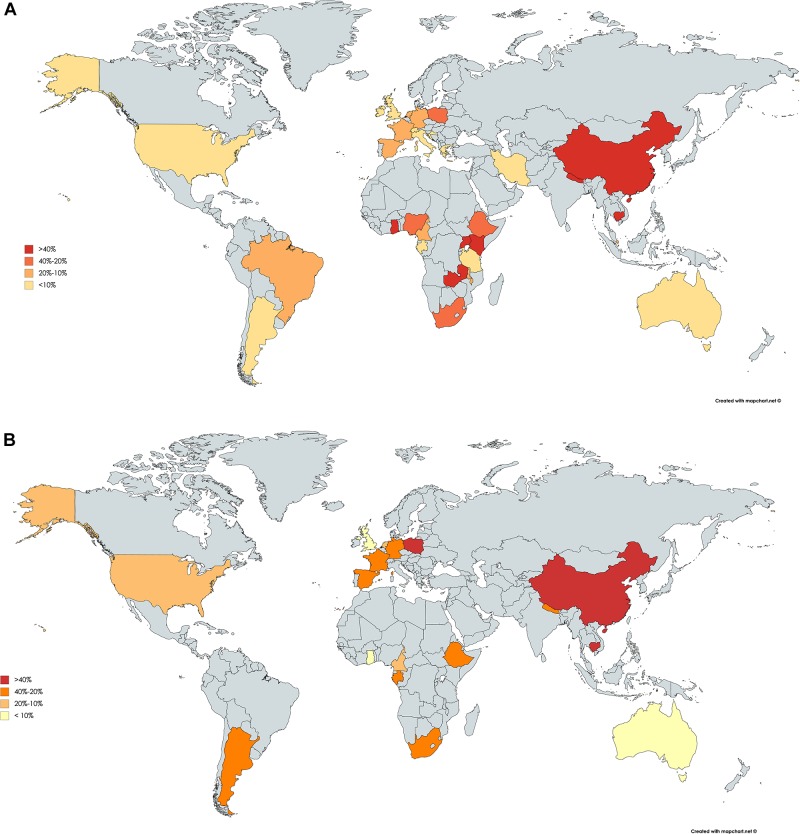
Worldwide seroprevalence of anti-IgG hepatitis E virus in HIV-infected patients **(A)**. Schematic representation of the distribution of hepatitis E IgG antibody seroprevalence. Worldwide seroprevalence of anti-IgG hepatitis E virus in HIV-infected patients using Wantai Diagnostics assay **(B)**. Maps created based on the data obtained in Supplementary Table S1.

Studies evaluating the seroprevalence of IgG anti-HEV in HIV-infected subjects were conducted on all continents (Figure 1). Higher seroprevalence was reported in Africa and Asia (exceeding 40% in the majority of countries), followed by continental European Union countries (20–10%) and, finally, the Americas and Oceania (<10%). Full details of the studies evaluating HEV seroprevalence in HIV-infected individuals are presented in Supplementary Table S1. Several studies evaluated HEV seroprevalence in HIV-specific populations. In this sense, three studies evaluated the HEV IgG prevalence in pregnant women with HIV in Africa, reporting a seroprevalence of 7.1% in Gabon (Caron et al., 2012), 7.4% in Malawi (Mancinelli et al., 2017), and 33.3% in Ethiopia (Abebe et al., 2017). Finally, the prevalence of HIV-infected patients who are candidates for liver and kidney transplants in the United States was 19.2% (Sherman et al., 2014). Nevertheless, when interpreting and comparing HEV seroprevalence data, it should be considered that the immune assays used for HEV antibody identification demonstrate different sensitivities (Aggarwal, 2013). These differences were noted in a recent meta-analysis that included studies conducted in Europe, with the main aim to evaluate the seroprevalence in different subsets of patients according to the serological assays employed (Hartl et al., 2016). In this study, the seroprevalence rates found in HIV-infected patients were 1.8, 3.75, 5.9, 9.26, 11.55, and 15.69%, depending on the assay used. Similarly, a study conducted in Germany that included 246 HIV-infected individuals reported that the seroprevalence strongly varied from 1.6 to 25.6%, depending on the anti-HEV assays used (Pischke et al., 2015).

Few studies have reported HEV seroincidence in HIV-infected patients. Two studies conducted in Spain showed HEV seroincidence rates of 2.4 and 6.5% in 1 year, respectively (Pineda et al., 2014; Rivero-Juarez et al., 2017a). In a study that enrolled HIV-infected patients from China, the annual HEV seroincidence was 15.4% (Zeng et al., 2017). Finally, another study that included HIV-infected pregnant women from Tanzania reported an annual seroincidence of 1% (Harritshøj et al., 2018).

#### Transmission and Risk Groups

The HEV transmission route varies depending on the viral genotype. HEV genotypes 1 and 2, which exclusively affect humans, are mainly transmitted by the consumption of fecal-contaminated water during the rainy season and are associated with flooding, as noted by the WHO (World Health Organization [Who], 2014). Similarly, a lack of hygienic measures, such as a lack of hand washing or the absence of proper sanitation, is an important risk factor for the acquisition of HEV in the general population (World Health Organization [Who], 2014). In a study conducted in Nigeria that enrolled HIV-infected patients, an inadequate toilet system (pit and bush) and water supply source (well and stream) were identified as major risk factors for HEV infection (Junaid et al., 2014). In contrast, HEV genotypes 3 and 4 as well as the less-common genotype 7 can infect both humans and a wide range of animals; thus, they constitute a zoonotic infectious disease (EFSA, 2017). In the general population, the main routes of transmission of these genotypes are the consumption of raw or undercooked meat (overall pork) and contact with infected animals (EFSA, 2017). In this sense, the HEV seroprevalence in HIV-infected individuals who eat raw/undercooked pork was 33% compared with 9% in those who not reported consumption in a study conducted in southwest England (Keane et al., 2012). In addition, one outbreak, which was linked to the consumption of wild boar meat in Spain, that involved HIV-infected patients has been recently reported (Rivero-Juarez et al., 2017c). Other factors associated with HEV seroprevalence in the general population, such as older age (Feldt et al., 2013; Rapicetta et al., 2013; Pineda et al., 2014; Rivero-Juarez et al., 2015b; Shrestha et al., 2017; Zeng et al., 2017; Zhou et al., 2018), geographical location or habitat (Renou et al., 2010; Feldt et al., 2013; Rapicetta et al., 2013; Junaid et al., 2014; Scotto et al., 2014; Yong et al., 2014; Rivero-Juarez et al., 2017a; Shrestha et al., 2017; Zeng et al., 2017), and male sex (Pineda et al., 2014; Zeng et al., 2017; Boon et al., 2018), have also been associated with HIV-infected patients. Consequently, the main route of HEV, including all viral genotypes, does not differ between the general and HIV populations.

**Table 1:** Studies evaluating HIV infection as a risk factor for hepatitis E virus infection, including comparator groups.

Study references	HIV population (n)	Comparator group (n)	HEV seroprevalence (%) HIV group	HEV seroprevalence (%) Comparator group	HIV infection identified as risk factor
Payne et al., 2013	146 MSM	135 MSM 141 Htx	7.5%	10.4% MSM 3.5% Htx	No
Keane et al., 2012	138	464 patients without history of CLD	9.4%	13.8%	No
Rapicetta et al., 2013	72	896 HD	19.4%	11.1%	Yes
Scotto et al., 2014					
Abebe et al., 2017	18 pregnant women	368 pregnant women	33.3%	31.5%	No
Bura et al., 2017b	244	246 HD	50.8%	49.6%	No
Madden et al., 2016	60	896 HD	23.3%	29.1%	No
Fainboim et al., 1999	484	1500 HD	6.6%	1.8%	Yes
Maylin et al., 2012	261	46 kidney Tx	1.5%	6.5%	No
Bura et al., 2017a	105	105 HD	0.95%	3.8%	No
Abravanel et al., 2017	300	600 HD	IgG: 38.7% IgM: 3.6%	IgG: 47.3% IgM: 3.8%	No
Boon et al., 2018	494	491 HD	46.4%	47.7%	No
Shrestha et al., 2017	459	581 HD	IgG: 39.4% IgM: 15.3%	IgG: 9.5% IgM: 4.4%	Yes
Junaid et al., 2014	80	190 HD 108 pregnant women 48 animal handlers	30%	44.7% healthy donors 41.6% pregnant women 58.3% animal handler	No
Taha et al., 2015	403	397 HD	12.9%	20.2%	No
Riveiro-Barciela et al., 2014	238	301 CLD patients 338 Liver Tx 296 Kidney Tx 200 HD	9.2%	4.9% CLD patients 9.4% Liver Tx 3.7% Kidney Tx 3.5% HD	Yes

*HIV, human immunodeficiency virus; n, number of subjects; HEV, hepatitis E virus; MSM, men who have sex with men; Htx, heterosexual; HD, healthy donors; HCV, hepatitis C virus; CLD, chronic liver disease; Liver Tx, liver transplant; Kidney Tx, kidney transplant.*

Several studies have evaluated whether HIV infection constitutes a risk factor for HEV infection (Table 1) (Fainboim et al., 1999; Keane et al., 2012; Maylin et al., 2012; Rapicetta et al., 2013; Payne et al., 2013; Junaid et al., 2014; Riveiro-Barciela et al., 2014; Scotto et al., 2014; Taha et al., 2015; Madden et al., 2016; Abebe et al., 2017; Abravanel et al., 2017; Bura et al., 2017a,b; Shrestha et al., 2017; Boon et al., 2018). Of these studies, only three matched the controls by age, sex and geographical area (Abravanel et al., 2017; Bura et al., 2017a; Boon et al., 2018). Consequently, HIV *per se* seems not to be a risk factor for HEV infection. Other not matched studies found differences in favor to healthy donors or HIV infected patients. A study conducted in Uganda that included 491 healthy blood donors and 494 HIV-infected patients showed no differences in terms of HEV IgG seroprevalence between both groups (47.7 and 46.4%) (Boon et al., 2018). Another study performed in Poland showed a relatively higher HEV seroprevalence in healthy donors (3.8%) than that in subjects infected with HIV (0.95%) (Bura et al., 2017a). Similarly, in a French study, the HEV IgG seroprevalence in healthy donors was higher than that in HIV-infected patients (47.3 and 38.7%) (Abravanel et al., 2017).

Specific conditions related to HIV infection have been evaluated as potential factors associated with HEV infection. First, HIV viremia seems to have no association with a higher risk for HEV infection (Jardi et al., 2012; Keane et al., 2012; Pineda et al., 2014; Rivero-Juarez et al., 2015b; Abravanel et al., 2017; Ferreira et al., 2018). Therefore, the rate of IgG HEV antibodies is similar among patients with detectable and undetectable HIV viral loads (Jardi et al., 2012; Keane et al., 2012; Pineda et al., 2014; Rivero-Juarez et al., 2015b; Abravanel et al., 2017; Ferreira et al., 2018). In the same way, the use and duration of antiretroviral therapy has not been associated with a higher HEV seroprevalence in different studies that have reported a comparable percentage between patients on antiretroviral therapy and those not using this type of therapy (Jardi et al., 2012; Keane et al., 2012; Feldt et al., 2013; Pineda et al., 2014; Rivero-Juarez et al., 2015b; Abravanel et al., 2017; Ferreira et al., 2018). Furthermore, one study did not find differences in HEV seroprevalence between HIV-1- and HIV-2-infected patients (Kaba et al., 2011). Finally, studies evaluating the association between CD4+ cell count and HEV seroprevalence present controversial results. The majority of these studies found a similar HEV seroprevalence between patients with a CD4+ cell count that was either higher or lower than 200 cells/mL (Kaba et al., 2011; Kenfak-Foguena et al., 2011; Jardi et al., 2012; Pineda et al., 2014; Bura et al., 2017b; Ferreira et al., 2018), or when a cut-off of 250 cells/mm^3^ was applied (Zeng et al., 2017). Nevertheless, there are studies that found a higher HEV seroprevalence in patients with a total CD4+ cell count higher than 200 cells/mL (Kenfak-Foguena et al., 2011), while other studies found the opposite effect, in that patients with a CD4+ count below 200 cells/mL showed a higher seroprevalence of IgG antibodies (Renou et al., 2010; Debes et al., 2016; Zhou et al., 2018).

Several observations have suggested that men who have sex with men (MSM) may be exposed to an increased risk for HEV transmission. In a study conducted in the United Kingdom (UK), the seroprevalence of HEV was compared among 146 HIV-infected MSM, 135 HIV-non-infected MSM, and 141 HIV-non-infected heterosexual men. The seroprevalence was similar between both MSM groups (7.5% vs. 10.4%; *p* = 0.4) but was higher than that found in heterosexual men (3.5%; *p* = 0.025) (Payne et al., 2013). Similarly, in an Italian study in which the HEV seroprevalence was evaluated in a large cohort of individuals that included HIV-infected patients, the seroprevalence found in MSM was higher than the seroprevalence reported in non-MSM (7.5% vs. 4.7%; *p* = 0.04) (Lanini et al., 2015). Finally, another Italian study analyzed the seroprevalence of HEV and hepatitis A virus (HAV) in a cohort of 636 MSM and compared the seroprevalence with that of a control group of 288 non-MSM (Greco et al., 2018). This study reported a higher seroprevalence for both HAV and HEV (42.8 and 10.2%) in the MSM group than that in the non-MSM group (29.2 and 5.2%). In contrast, a recent study conducted in Taiwan during an HAV outbreak including 3,293 HIV-infected patients, majoritarian MSM, the seroprevalence and seroincidence of HEV infection did not differ between sexual risk practice or underlying infection (HAV, HCV, or Syphilis) (Lin et al., 2019). Interestingly, in only 1 of the 23 HEV seroconversion documented in the study also showed seroconversion for HAV. For this reason, the fact that HIV-infected MSM might be a population that is at higher risk for HEV acquisition need to be clarify.

### Diagnosis of HEV Infection in HIV-Infected Patients

The virological markers for the diagnosis of HEV infection comprise viral components (HEV RNA and HEV Antigen [HEV Ag]) and products related to the host immune response (anti-HEV specific antibodies of IgA, IgG, and IgM classes) (Aggarwal, 2013). Typically, in the general population, after an incubation period between 2 and 6 weeks, viral RNA and HEV Ag are detectable in the blood, urine, and feces (Zhao and Wang, 2016). After 4–6 weeks of infection, HEV-RNA is usually undetectable in the blood but can remain detectable in the feces for several weeks (Aggarwal, 2013). The immune response follows a typical pattern of seroconversion with an initial and transient increase in IgM that leads to a sustained IgG response. Anti-HEV IgM antibodies are detected only during the acute phase and remain detectable up to the 5th month after infection, making these antibodies the best serological markers for the diagnosis of acute HEV infection. Furthermore, IgG antibodies can be detected very close in time to the detection of IgM antibodies, and they remain detectable for more than 10 years; thus, they may be used to establish past exposure to HEV (Aggarwal, 2013). Finally, IgA antibodies can also be detected during the acute phase of HEV infection, but their use in the diagnosis of HEV infection is controversial (Aggarwal, 2013).

Studies have evaluated the prevalence of HEV IgM antibodies in HIV-infected patients, and they have found a good value for the diagnosis of acute/recent HEV infection in this population (Abravanel et al., 2017; Shrestha et al., 2017). Furthermore, several studies have shown that the absence of HEV-IgM antibodies is correlated with the absence of HEV RNA in HIV-infected patients (Ramezani et al., 2013; Harritshøj et al., 2018), even in patients with severe immunosuppression (Nouhin et al., 2015; Rivero-Juarez et al., 2015a). In addition, several studies of HEV infection show that HEV-IgM positivity coincides with the first detectable HEV viral load (Renou et al., 2010; Sellier et al., 2011; Bouamra et al., 2013; Robbins et al., 2014; Abravanel et al., 2017). In contrast, there is evidence that the use of HEV IgM to diagnose acute HEV infection in this population has limited value because studies have shown that HIV-infected patients may have a delayed immune response or may lack an immune response against acute HEV infection. One of these studies, in which three cases of acute HEV infection were described, showed that all cases lacked HEV IgM positivity and seroconversion over time; additionally, they all showed a CD4+ count higher than 200 cells/mm^3^ (Kuniholm et al., 2016). Similarly, another study that included 5 cases of acute HEV infection diagnosed by HEV RNA found that HEV IgM could only be detected at diagnosis in two subjects (Rivero-Juarez et al., 2015b), and three of these patients lacked HEV IgM positivity, with a CD4+ cell count higher than 200 cells/mm^3^. Similarly, in a study of 10 cases of acute HEV infection in Brazil, none of the patients presented with HEV HEV IgM, or HEV IgG antibodies (Salvio et al., 2018). Of the 9 patients with an available CD4+ count, only one showed a CD4+ cell count lower than 200 cells/mL (Salvio et al., 2018). These data show that the lack of HEV IgM during the acute phase may not be limited to those patients with severe immunosuppression. For these reasons, in HIV-infected patients, the application of HEV IgM alone may not be sufficient to exclude the diagnosis of acute HEV infection, and it is mandatory to include a direct diagnosis procedure such as HEV RNA (European Association for the Study of the Liver [EASL], 2018; Rivero-Juarez et al., 2018).

After the occurrence of the acute phase in HIV-infected patients, HEV can persist and may develop into a chronic infection, which is defined as the persistence of HEV RNA for more than 3 months (European Association for the Study of the Liver [EASL], 2018; Rivero-Juarez et al., 2018). In these patients, IgM and IgG antibody seroconversion is usually absent or can be detected intermittently (Colson et al., 2009; Dalton et al., 2009; Kaba et al., 2011; Kenfak-Foguena et al., 2011; Andersson et al., 2013; Jagjit, Singh et al., 2013; Neukam et al., 2013; Ingiliz et al., 2016; Kuniholm et al., 2016; Todesco et al., 2017). On the other hand, it has been described that in HIV infected patients HEV reinfection may occur (Rivero-Juarez et al., 2017b). The serological pattern of HEV reinfection is characterized by the presence of HEV RNA with positivity to HEV IgG antibodies and the persistent absence of HEV IgM (European Association for the Study of the Liver [EASL], 2018; Rivero-Juarez et al., 2018). Thus, the only marker that is indicative of these two virological situations is the use of methods evaluating HEV RNA.

### Clinical Impact of HEV Infection in HIV-Infected Patients

Acute HEV infection usually occurs as mild-severity acute hepatitis in both the general and HIV-infected populations. In several patients, hospitalization is necessary (Kamar et al., 2012), with an associated mortality of up to 8.7%, which varies depending on the comorbidities of the patients affected (Péron et al., 2006; Dalton et al., 2008). In patients with underlying chronic liver disease, acute HEV infection is associated with a high overall mortality rate in patients from low-income countries who are infected with HEV genotypes 1 and 2 (Kumar Acharya et al., 2007). Several studies have evaluated the prevalence of acute HEV infection in HIV-infected patients with acute increases in transaminases. In a study conducted in Scotland of 99 HIV-infected patients, the prevalence of acute HEV was 1.06% (Bradley-Stewart et al., 2015). Another study performed in France found that acute HEV infection was a cause of unexplained elevated transaminases in one patient of the 108 HIV-infected subjects evaluated (0.9%) (Sellier et al., 2011). In the United States, among 458 HIV-infected patients who were United States military beneficiaries, evidence of acute HEV infection was detected in 4% of 194 HIV-infected persons with an episode of increased transaminase levels (Crum-Cianflone et al., 2012). In contrast, a study that included 256 HIV-infected patients at follow-up in the Netherlands did not find any cases of acute HEV (Hassing et al., 2014). For this reason, clinical guidelines recommend excluding HEV in cases of acute hepatitis (European Association for the Study of the Liver [EASL], 2018; Rivero-Juarez et al., 2018). Until today, no hepatic decompensation among HIV cirrhotic patients has been reported.

Furthermore, there is increasing knowledge regarding extrahepatic manifestations that are linked to acute HEV infection, highlighting neurological injury, renal injury, cryoglobulinemia, pancreatitis, and hematological disorders (Dalton et al., 2016; Pischke et al., 2017). Although there are no specific studies that describe the course of HEV infection with extrahepatic manifestations in HIV-infected patients, the cases reported in this population suggest a similar clinical pattern to that reported in a non-HIV-infected population during the acute phase (Bouamra et al., 2013; Robbins et al., 2014; Kuniholm et al., 2016). Nevertheless, in a series of cases that included both immunocompetent and immunocompromised patients (with only three HIV-infected patients in this group), it was suggested that acute hepatitis-related symptoms and neurological manifestations may occur at lower frequencies in immunocompromised patients (Abravanel et al., 2018).

**Table 2:** Cases of chronic hepatitis E virus infection in HIV-infected patients.

Case references	Gender	Age	CD4+ cells count	HEV anti IgM/IgG	HEV genotype	Duration of HEV viremia (months)	Outcome	Comment
Dalton et al., 2009	Male	48	<100	Pos/Pos	3a	24	Treatment initiation	
Colson et al., 2009	Male	50	<100	Neg/Neg	3	12	Not reported	
Jagjit, Singh et al., 2013	Male	45	54	Neg/Neg	3a	132	Treatment initiation	
Kaba et al., 2011	Male	44	40	Pos/Neg	3f	10	Deceased	Diagnosis of chronic infection coincides with treatment time for non-Hodgkin’s lymphoma therapy
Kenfak-Foguena et al., 2011	Male	46	34	NT/Pos	3b	36	Spontaneous viral clearance	Viral clearance after CD4+ count increases
Kenfak-Foguena et al., 2011	Male	59	50	NT/Neg	3c	At least 6	Spontaneous viral clearance	
Andersson et al., 2013	Male	42	37	Neg/Neg	3	36	Spontaneous viral clearance	Clearance after HAART initiation and CD4+ count restoration
Neukam et al., 2013	Male	47	<200	Pos/Neg	3	36	Treatment initiation	
Neukam et al., 2013	Male	53	<200	Neg/Pos	3	60	Treatment initiation	
Kuniholm et al., 2016	Female	–	<200	Neg/Neg	3a	36	Not reported	
Ingiliz et al., 2016	Male	47	<200	Pos/Pos	3	48	Treatment initiation	
Todesco et al., 2017	Male	59	<100	NT/NT	3i	48	Treatment initiation	

*CD4+, lymphocytes CD4+; HEV, hepatitis E virus; IgM, immunoglobulin M; IgG, immunoglobulin G; Pos, positive; Neg, negative; NT, non-tested.*

**Table 3:** Treatment of acute and chronic HEV in HIV-infected patients.

Phase	References	HEV genotype	Regimen	Outcome
Acute	Abravanel et al., 2017	3f	RBV 24 weeks	Treatment induced viral clearance
Acute	Robbins et al., 2014	3c	RBV 24 weeks 1,200 mg	Treatment induced viral clearance
Acute	Bouamra et al., 2013	3c	RBV 24 weeks 1,200 mg	Treatment induced viral clearance
Chronic	Dalton et al., 2011	3a	Peg-IFN 24 and 6 weeks of Peg-IFN/RBV	Treatment induced viral clearance
Chronic	Jagjit, Singh et al., 2013	3a	Peg-IFN 24 weeks	Treatment induced viral clearance
Chronic	Neukam et al., 2013	3	RBV 24 weeks 1,200 mg	Viral relapse
Chronic	Neukam et al., 2013	3	RBV 24 weeks 1,000 mg	Viral relapse
Chronic	Ingiliz et al., 2016	3	RBV 20 weeks 800 mg	Treatment induced viral clearance
Chronic	Todesco et al., 2017	3i	1° Peg-IFN/RBV 12 weeks 2° SOF/RBV 12 weeks	1° Viral relapse 2° Viral relapse

*HEV, hepatitis E virus; RBV, ribavirin; Peg-IFN, pegylated interferon; SOF, sofosbuvir.*

Furthermore, 4–6 weeks after the appearance of clinical symptoms, the infection is usually self-limited and does not need therapy. However, in immunosuppressed patients with various causes, the infection can evolve into a chronic infection (European Association for the Study of the Liver [EASL], 2018; Rivero-Juarez et al., 2018). This development usually occurs with HEV genotypes 3 and 4 and is characterized by a rapid progression to liver cirrhosis and the persistence of changes at the transaminase level (Neukam et al., 2013). The prevalence of chronic HEV infection among HIV-infected patients is rare, with an estimated prevalence between 0 and 0.5% (Madejón et al., 2009; Pischke et al., 2010; Renou et al., 2010; Sellier et al., 2011; Maylin et al., 2012; Scotto et al., 2014; Sherman et al., 2014; Nouhin et al., 2015; Rivero-Juarez et al., 2015b; Abravanel et al., 2017; Ferreira et al., 2018). In a Spanish study, the prevalence of chronic HEV infection among HIV-infected patients with unexplained increases in liver stiffness was 0.5% (Rivero-Juárez et al., 2013). In two cohorts of HIV-infected patients from France and the United States, the prevalence of chronic HEV infection was 0.5 and 0.05%, respectively (Kaba et al., 2011; Kuniholm et al., 2016). Finally, in a study conducted in Switzerland that included 735 HIV-infected patients with persistent ALT levels, the prevalence of chronic HEV infection was 0.13% (Kenfak-Foguena et al., 2011). Currently, 12 cases of chronic HEV infection have been reported in HIV-infected patients (Table 2) (Colson et al., 2009; Dalton et al., 2009; Kaba et al., 2011; Kenfak-Foguena et al., 2011; Andersson et al., 2013; Jagjit, Singh et al., 2013; Neukam et al., 2013; Ingiliz et al., 2016; Kuniholm et al., 2016; Todesco et al., 2017). All HIV cases have a CD4+ cell count lower than 200 cells/mm^3^, which is the only risk factor associated with the development of chronic HEV infection in this population.

### Treatment of Acute and Chronic HEV Infection in HIV-Infected Patients

Most cases of acute HEV infection are self-limited without the need for the implementation of therapy in both the general and HIV-infected populations. However, in several subsets of patients, such as patients with underlying chronic liver disease or acute liver failure, acute HEV infection is associated with a higher risk of complications and a worse prognosis (Kumar Acharya et al., 2007). Currently, there are no specific therapies available against acute HEV infection (European Association for the Study of the Liver [EASL], 2018; Rivero-Juarez et al., 2018). The evidence available regarding the use of ribavirin (RBV) in the HIV-infected population for the treatment of acute HEV infection is limited to three cases of infection with genotype 3 (Table 3), all of which were successfully treated with RBV monotherapy for 12 or 24 weeks (Bouamra et al., 2013; Robbins et al., 2014; Abravanel et al., 2017).

The development of chronic HEV infection is associated with immunosuppression; thus, measures aimed at restoring the immune response may induce the clearance of HEV (European Association for the Study of the Liver [EASL], 2018; Rivero-Juarez et al., 2018). In this sense, two cases of chronic HEV infection in HIV-infected patients were self-limiting after the initiation of antiretroviral therapy and the suppression of HIV viral load (Table 2) (Kenfak-Foguena et al., 2011; Andersson et al., 2013). Relatively large amounts of evidence regarding the therapeutic options for chronic HEV infection are available for liver transplant patients (McPherson et al., 2018). In contrast, evidence in HIV-infected patients is scarce and limited to the description of 6 cases (Table 3). Three cases have reported treatment with RBV monotherapy (Neukam et al., 2013; Ingiliz et al., 2016), and two of these patients experienced viral relapse after the cessation of therapy [63]. Another three cases reported the combination of pegylated interferon with RBV for 12 and 24 weeks of treatment (Dalton et al., 2011; Jagjit, Singh et al., 2013; Todesco et al., 2017), and two of these patients attained sustained viral clearance (Dalton et al., 2011; Jagjit, Singh et al., 2013). Finally, the *in vitro* antiviral activity of sofosbuvir against HEV has been demonstrated (Dao Thi et al., 2016). Nevertheless, the use of this drug in the transplant population for the treatment of chronic HEV has not been demonstrated to have efficacy in eliminating the virus (van der Valk et al., 2017; Todesco et al., 2018). In the HIV-infected population, the use of sofosbuvir in combination with RBV for 12 weeks has been evaluated in only one case (Todesco et al., 2017). This patient experienced viral relapse after the completion of therapy.

### Prevention of HEV in HIV-Infected Patients

The most effective preventative measure for HEV infection is to avoid contact with the source of infection, such as avoiding the consumption of raw/undercooked food where HEV has been isolated or the consumption of unchlorinated contaminated water in low-income countries (European Association for the Study of the Liver [EASL], 2018; Rivero-Juarez et al., 2018). Treating meat at a temperature of 70°C for 30 min has been shown to strongly inhibit HEV activity (Johne et al., 2016). For contaminated milk, thermal treatment at 100°C has shown complete inactivation of the virus (Huang et al., 2016). For contaminated water, a chlorine dose of 5 mg/L for 15 min appears to be sufficient to reduce the HEV viral load; nevertheless, it may be necessary to increase the chlorine dose if water contains solid material (World Health Organization [Who], 2014; EFSA, 2017). Clinical guidelines recommend that immunocompromised patients, such HIV-infected patients with a CD4+ cell count below 200 cells/mm^3^ and those with chronic liver disease including HIV-infected patients with coinfection with HCV or HBV, should avoid the consumption of raw or undercooked meat and shellfish due to the risk of developing a serious or even fatal course of HEV infection (European Association for the Study of the Liver [EASL], 2018; Rivero-Juarez et al., 2018).

Currently, there is only one vaccine available for the prevention of HEV infection, which is a recombinant vaccine against genotype 1 that has demonstrated high protection of up to 5 years for people over 16 years of age, with potential protection of up to 30 years (Zhang et al., 2015; Su et al., 2017). Nevertheless, the application of this vaccine is currently limited to China, following the position of the WHO (World Health Organization [Who], 2015). This position is supported by the fact that there are no clinical trials evaluating the safety and efficacy of this vaccine in populations susceptible to a worsened prognosis of the disease (HIV-infected patients, transplant recipients, pregnant women, and patients with underlying chronic liver disease) and because of only demonstrated efficacy in preventing HEV genotype 1 infection. For this reason, the WHO recommends that vaccination should be considered individually in people who plan to travel to an area where an epidemic is occurring (e.g., aid workers and health workers).

Finally, in immunosuppressed patients, such as transplant recipients and HIV-infected patients (Abravanel et al., 2014; Rivero-Juarez et al., 2017b), HEV reinfection has been described. Furthermore, in immunocompetent patients, an IgG antibody concentration of 2.5 WHO units/mL could protect against reinfection (Zhang et al., 2015); in immunocompromised patients, this titre can be increased up to 7 WHO units/mL (Abravanel et al., 2014). Nevertheless, there are no data regarding the minimum protective titre of IgG antibodies in HIV-infected patients. This fact reinforces the recommendation that preventive measures should be applied in immunosuppressed HIV-infected patients, even in the presence of IgG antibodies. Furthermore, due to the risk of reinfection and the lack of diagnostic value of IgM antibodies at this point, annual testing for HEV-RNA in HIV immunocompromised patients is currently recommended in clinical guidelines (European Association for the Study of the Liver [EASL], 2018; Rivero-Juarez et al., 2018).

## Conclusion

Despite the increasing knowledge about the pathogenesis, epidemiology and clinical impact of HEV infection, several major factors are faced by HIV-infected patients. First, HIV infection seems not to be a risk factor for HEV acquisition. Nevertheless, the risk population for HIV infection, such as MSM, may also have a higher risk for HEV infection. A better understanding of this risk significantly increases the awareness of prevention in this population. Second, in recent years, HEV-associated extrahepatic manifestations have been described, with special emphasis on acute neurological injury. However, there is a lack of clinical data on these extrahepatic manifestations in HIV-infected patients; thus, there is no evidence on the frequency, clinical course and management that can support any special recommendations. Third, therapy for both acute and chronic HEV infections is suboptimal, with very limited evidence for combination and posology therapies in the HIV-infected population. The use of direct-acting viral drugs, such as sofosbuvir, against other viral infections has shown a lack of efficacy in clinical practice in terms of chronic HEV infection in both the general and HIV populations. Therefore, evaluating alternative therapeutic options is a priority. Finally, the only approved vaccine for preventing HEV infection has a lack of data regarding safety and efficacy in HIV-infected patients. Thus, before vaccination of these patients, there is a need for data as well as the overall evaluation of the persistence of protected antibodies over time in severely immunocompromised HIV patients.

## Author Contributions

AR-J drafted the manuscript and performed the research strategy with the support of all authors. All authors approved the final version of the manuscript.

## Conflict of Interest Statement

AR-J received public funding for this research by Fundación para la Investigación en Salud (FIS) of the Instituto de Salud Carlos III. Additionally, he received private research support by AbbVie y ViiV Healthcare. He received payment for consultation by Roche Diagnostics, Gilead Sciences, and Bristol-Myers Squibb and payment for lectures, including service on speakers bureaus from Bristol-Myers Squibb, ViiV Healthcare, Janssen Cilag, MSD, y Roche Diagnostics, AbbVie, and Gilead Sciences. AR has received public funds for this research by Fundación para la Investigación en Salud (FIS) of the Instituto de Salud Carlos III. Additionally, he received private research support by AbbVie, ViiV Healthcare, Gilead Sciences, Bristol-Myers Squibb, and Janssen Cilag. He has received payment for consultancy by Gilead Sciences, Bristol-Myers Squibb, Janssen Cilag, AbbVie, and ViiV Healthcare. MF has received public funds for this research by Fundación Progreso y Salud of the Junta de Andalucía. The remaining author declares that the research was conducted in the absence of any commercial or financial relationships that could be construed as a potential conflict of interest.
