# Supplementary material for: Hepatitis E Infection in HIV-Infected Patients
Source: Front Microbiol. 2019 Jun 26;10:1425. doi: 10.3389/fmicb.2019.01425 (PMC6608679; doi:10.3389/fmicb.2019.01425)
Supplement: Supplementary file 1 [file Table_1.docx]

**Supplementary Table 1.** Studies evaluating IgG anti-HEV seroprevalence in HIV infected patients. These data have been used to construct the prevalence map.

| **Country** | **EIA assay employed** | **N** | **IgG+ patients** | **Reference** |
| --- | --- | --- | --- | --- |
| Argentina | Wantai Diagnostic Assay | 28 | 10 | [1] |
|  | Diapro | 204 | 15 | [2] |
|  | Abbott laboratories | 484 | 32 | [3] |
| Australia | Wantai Diagnostic Assay | 191 | 12 | [4] |
| Brazil | RecomLine Kits | 354 | 38 | [5] |
| Cambodia | Wantai Diagnostic Assay | 170 | 73 | [6] |
| Cameroon | Wantai Diagnostic Assay | 289 | 41 | [7] |
| China | Wantai Diagnostic Assay | 770 | 342 | [8] |
|  | Wantai Diagnostic Assay | 639 | 252 | [9] |
| Croatia | RecomLine Kits | 88 | 1 | [10] |
| Etiopia | Wantai Diagnostic Assay | 18 | 6 | [11] |
| France | Wantai Diagnostic Assay | 300 | 116 | [12] |
|  | Adaltis | 242 | 15 | [13] |
|  | Adaltis | 184 | 8 | [14] |
|  | Adaltis | 108 | 4 | [15] |
|  | Adaltis | 261 | 4 | [16] |
| Gabon | Wantai Diagnostic Assay | 257 | 9 | [17] |
|  | GeneLabs | 183 | 13 | [18] |
| Germany | Wantai Diagnostic Assay | 246 | 63 | [19] |
|  | MP Diagnostic Assay | 246 | 4 | [19] |
|  | Abbott laboratories | 123 | 6 | [20] |
| Ghana | Wantai Diagnostic Assay | 402 | 182 | [7] |
| Greece | Adaltis | 243 | 18 | [21] |
| Holland | Wantai Diagnostic Assay | 256 | 27 | [22] |
| Iran | Diapro | 100 | 10 | [23] |
|  | Diapro | 158 | 12 | [24] |
| Italy | DSI | 72 | 14 | [25] |
|  | DSI | 509 | 34 | [26] |
|  | Diapro | 55 | 3 | [27] |
|  | DSI | 100 | 2 | [28] |
| Kenya | DSI | 34 | 27 | [29] |
| Malaysia | Abbott laboratories | 145 | 15 | [30] |
| Malawi | Wantai Diagnostic Assay | 403 | 52 | [31] |
|  | Diapro | 268 | 20 | [32] |
| Nepal | Wantai Diagnostic Assay | 459 | 181 | [33] |
| Nigeria | Diagnosis Automation Inc | 80 | 24 | [34] |
| Poland | Wantai Diagnostic Assay | 244 | 124 | [35] |
|  | EUROIMMUN | 105 | 1 | [36] |
| U.K. | RecomLine Kits | 94 | 1 | [37] |
|  | Wantai Diagnostic Assay | 146 | 11 | [38] |
|  | Wantai Diagnostic Assay | 138 | 13 | [39] |
| South Africa | Wantai Diagnostic Assay | 60 | 14 | [40] |
| Spain | Wantai Diagnostic Assay | 613 | 161 | [41] |
|  | Wantai Diagnostic Assay | 894 | 189 | [42] |
|  | MP Diagnostic Assay | 225 | 35 | [43] |
|  | Diapro | 178 | 18 | [44] |
|  | Bioelisa | 238 | 22 | [45] |
|  | MP Diagnostic Assay | 238 | 22 | [46] |
|  | Diapro | 488 | 45 | [47] |
| Switzerland | Adaltis | 735 | 19 | [48] |
| Tanzania | Nordic BioSite | 200 | 16 | [49] |
| Uganda | In house using WHO 95/584 | 494 | 229 | [50] |
| U.S.A. | Nizhniy Novgorod | 194 | 13 | [51] |
|  | Wantai Diagnostic Assay | 166 | 32 | [52] |
| Taiwan | RecomLine Kits | 3293 | 123 | [54] |
| Zambia | Fortress | 31 | 22 | [53] |

**Legend:** Enzime immunoassay (EAI); number of patients tested (N); positive immunoglobulin G anti-HEV (IgG+); United Kingdom (U.K); United State of America (U.S.A.)

**Table references**

[1] Munné MS, Altabert NR, Otegui M LO, Vladimirsky SN, Moreiro R, Espul MP, Espul C, Manzur A, Soto SS, Brajterman LS, González JE. Updating the knowledge of hepatitis E: new variants and higher prevalence of anti-HEV in Argentina. Ann Hepatol. 2014 Sep-Oct;13(5):496-502.

[2] Debes JD, Martínez Wassaf M, Pisano MB, Isa MB, Lotto M, Marianelli LG, Frassone N, Ballari E, Bohjanen PR, Hansen BE, Ré V. Increased Hepatitis E Virus Seroprevalence Correlates with Lower CD4+ Cell Counts in HIV-Infected Persons in Argentina. PLoS One. 2016 Jul 28;11(7):e0160082.

[3] Fainboim H, González J, Fassio E, Martínez A, Otegui L, Eposto M, Cahn P, Marino R, Landeira G, Suaya G, Gancedo E, Castro R, Brajterman L, Laplumé H. Prevalence of hepatitis viruses in an anti-human immunodeficiency virus-positive population from Argentina. A multicentre study. J Viral Hepat. 1999 Jan;6(1):53-7.

[4] Yong MK, Paige EK, Anderson D, Hoy JF. Hepatitis E in Australian HIV-infected patients: an under-recognised pathogen? Sex Health. 2014 Sep;11(4):375-8.

[5] Ferreira AC, Gomes-Gouvêa MS, Lisboa-Neto G, Mendes-Correa MCJ, Picone CM, Salles NA, Mendrone-Junior A, Carrilho FJ, Pinho JRR. Serological and molecular markers of hepatitis E virus infection in HIV-infected patients in Brazil. Arch Virol. 2018 Jan;163(1):43-49.

[6] Nouhin J, Barennes H, Madec Y, Prak S, Hou SV, Kerleguer A, Kim S, Pean P, Rouet F. Low frequency of acute hepatitis E virus (HEV) infections but high past HEV exposure in subjects from Cambodia with mild liver enzyme elevations, unexplained fever or immunodeficiency due to HIV-1 infection. J Clin Virol. 2015 Oct;71:22-7.

[7] Feldt T, Sarfo FS, Zoufaly A, Phillips RO, Burchard G, van Lunzen J, Jochum J, Chadwick D, Awasom C, Claussen L, Drosten C, Drexler JF, Eis-Hübinger AM. Hepatitis E virus infections in HIV-infected patients in Ghana and Cameroon. J Clin Virol. 2013 Sep;58(1):18-23.

[8] Zhou S, Ren L, Xia X, Miao Z, Huang F, Li Y, Zhu M, Xie Z, Xu Y, Qian Y, Pan Q, Wang K. Hepatitis E virus infection in HIV-infected patients: A large cohort study in Yunnan province, China. J Med Virol. 2018 Jun;90(6):1121-1127.

[9] Zeng H, Wang L, Liu P, Liao L, Wang L, Shao Y. Seroprevalence of hepatitis E virus in HIV-infected patients in China. AIDS. 2017 Sep 10;31(14):2019-2021.

[10] Ðaković Rode O, Jemeršić L, Brnić D, Pandak N, Mikulić R, Begovac J, Vince A. Hepatitis E in patients with hepatic disorders and HIV-infected patients in Croatia: is one diagnostic method enough for hepatitis E diagnosis? Eur J Clin Microbiol Infect Dis. 2014 Dec;33(12):2231-6.

[11] Abebe M, Ali I, Ayele S, Overbo J, Aseffa A, Mihret A. Seroprevalence and risk factors of Hepatitis E Virus infection among pregnant women in Addis Ababa, Ethiopia. PLoS One. 2017 Jun 26;12(6):e0180078.

[12] Abravanel F, Lhomme S, Fougère M, Saune K, Alvarez M, Péron JM, Delobel P, Izopet J. HEV infection in French HIV-infected patients. J Infect. 2017 Mar;74(3):310-313.

[13] Renou C, Lafeuillade A, Cadranel JF, Pavio N, Pariente A, Allègre T, Poggi C, Pénaranda G, Cordier F, Nicand E; ANGH. Hepatitis E virus in HIV-infected patients. AIDS. 2010 Jun 19;24(10):1493-9.

[14] Kaba M, Richet H, Ravaux I, Moreau J, Poizot-Martin I, Motte A, Nicolino-Brunet C, Dignat-George F, Ménard A, Dhiver C, Brouqui P, Colson P. Hepatitis E virus infection in patients infected with the human immunodeficiency virus. J Med Virol. 2011 Oct;83(10):1704-16.

[15] Sellier P, Mazeron MC, Tesse S, Badsi E, Evans J, Magnier JD, Sanson-Le-Pors MJ, Bergmann JF, Nicand E. Hepatitis E virus infection in HIV-infected patients with elevated serum transaminases levels. Virol J. 2011 Apr 15;8:171.

[16] Maylin S, Stephan R, Molina JM, Peraldi MN, Scieux C, Nicand E, Simon F, Delaugerre C. Prevalence of antibodies and RNA genome of hepatitis E virus in a cohort of French immunocompromised. J Clin Virol. 2012 Apr;53(4):346-9.

[17] Bivigou-Mboumba B, Rouet F, Mouinga-Ondeme A, Deleplancque L, Sica J, Ndjoyi-Mbiguino A, Njouom R, François-Souquière S. Hepatitis B, C, and E infection among HIV-infected patients in Franceville, Gabon: retrospective cross-sectional study. Med Sante Trop. 2017 Aug 1;27(3):274-280.

[18] Caron M, Bouscaillou J, Kazanji M. Acute risk for hepatitis E virus infection among HIV-1-positive pregnant women in central Africa. Virol J. 2012 Oct 31;9:254.

[19] Pischke S, Schwarze-Zander C, Bremer B, Lehmann P, Wiegand SB, Gisa A, Behrendt P, Strassburg CP, Manns MP, Wedemeyer H, Rockstroh JK.Hepatitis E Virus Seroprevalence Rate in HIV-Infected Patients in Germany: A Comparison of Two Commercial Assays. Intervirology. 2015;58(5):283-7.

[20] Pischke S, Ho H, Urbanek F, Meyer-Olsen D, Suneetha PV, Manns MP, Stoll M, Wedemeyer H. Hepatitis E in HIV-positive patients in a low-endemic country. J Viral Hepat. 2010 Aug;17(8):598-9.

[21] Politou M, Boti S, Androutsakos T, Valsami S, Pittaras T, Kapsimali V. Seroprevalence of hepatitis E in HIV infected patients in Greece. J Med Virol. 2015 Sep;87(9):1517-20.

[22] Hassing RJ, van der Eijk AA, Lopes VB, Snijdewind IJ, de Man RA, Pas SD, van der Ende ME. Hepatitis E prevalence among HIV infected patients with elevated liver enzymes in the Netherlands. J Clin Virol. 2014 Aug;60(4):408-10.

[23] Ramezani A, Velayati AA, Khorami-Sarvestani S, Eslamifar A, Mohraz M, Banifazl M, Bidari-Zerehpoosh F, Yaghmaei F, McFarland W, Foroughi M, Keyvani H, Mostafavi E, Aghakhani A. Hepatitis E virus infection in patients infected with human immunodeficiency virus in an endemic area in Iran. Int J STD AIDS. 2013 Oct;24(10):769-74.

[24] Joulaei H, Rudgari O, Motazedian N, Gorji-Makhsous S. Hepatitis E virus seroprevalence in HIV positive individuals in Shiraz, Southern Iran. Iran J Microbiol. 2015 Apr;7(2):103-8.

[25] Rapicetta M, Monarca R, Kondili LA, Chionne P, Madonna E, Madeddu G, Soddu A, Candido A, Carbonara S, Mura MS, Starnini G, Babudieri S. Hepatitis E virus and hepatitis A virus exposures in an apparently healthy high-risk population in Italy. Infection. 2013 Feb;41(1):69-76.

[26] Scotto G, Grisorio B, Filippini P, Ferrara S, Massa S, Bulla F, Martini S, Filippini A, Tartaglia A, Lo Muzio L, Fazio V. Hepatitis E virus co-infection in HIV-infected patients in Foggia and Naples in southern Italy. Infect Dis (Lond). 2015;47(10):707-13.

[27] Lanini S, Garbuglia AR, Lapa D, Puro V, Navarra A, Pergola C, Ippolito G, Capobianchi MR. Epidemiology of HEV in the Mediterranean basin: 10-year prevalence in Italy. BMJ Open. 2015 Jul 14;5(7):e007110.

[28] Scotto G, Martinelli D, Centra M, Querques M, Vittorio F, Delli Carri P, Tartaglia A, Campanale F, Bulla F, Prato R, Fazio V. Epidemiological and clinical features of HEV infection: a survey in the district of Foggia (Apulia, Southern Italy). Epidemiol Infect. 2014 Feb;142(2):287-94.

[29] Furukawa NW, Teshale EH, Cosmas L, Ochieng M, Gikunju S, Fields BS, Montgomery JM. Serologic evidence for hepatitis E virus infection among patients with undifferentiated acute febrile illness in Kibera, Kenya. J Clin Virol. 2016 Apr;77:106-8.

[30] Ng KP, He J, Saw TL, Lyles CM. A seroprevalence study of viral hepatitis E infection in human immunodeficiency virus type 1 infected subjects in Malaysia. Med J Malaysia. 2000 Mar;55(1):58-64.

[31] Taha TE, Rusie LK, Labrique A, Nyirenda M, Soko D, Kamanga M, Kumwenda J, Farazadegan H, Nelson K, Kumwenda N. Seroprevalence for Hepatitis E and Other Viral Hepatitides among Diverse Populations, Malawi. Emerg Infect Dis. 2015 Jul;21(7):1174-82.

[32] Mancinelli S, Pirillo MF, Liotta G, Andreotti M, Jere H, Sagno JB, Amici R, Marazzi MC, Vella S, Palombi L, Giuliano M. Hepatitis E virus infection in HIV-infected pregnant women and their children in Malawi. Infect Dis (Lond). 2017 Sep;49(9):708-711.

[33] Shrestha A, Adhikari A, Bhattarai M, Rauniyar R, Debes JD, Boonstra A, Lama TK, Al Mahtab M, Butt AS, Akbar SMF, Aryal N, Karn S, Manandhar KD, Gupta BP. Prevalence and risk of hepatitis E virus infection in the HIV population of Nepal. Virol J. 2017 Nov 21;14(1):228.

[34] Junaid SA, Agina SE, Abubakar KA. Epidemiology and associated risk factors of hepatitis e virus infection in plateau state, Nigeria. Virology (Auckl). 2014 May 27;5:15-26

[35] Bura M, Łagiedo M, Michalak M, Sikora J, Mozer-Lisewska I. Hepatitis E virus IgG seroprevalence in HIV patients and blood donors, west-central Poland. Int J Infect Dis. 2017 Aug;61:20-22.

[36] Bura M, Bukowska A, Bura A, Michalak M, Mozer-Lisewska I. Hepatitis E virus antibodies in HIV-infected patients and blood donors from western Poland: A preliminary report. Adv Clin Exp Med. 2017 Jul;26(4):577-579.

[37] Bradley-Stewart AJ, Jesudason N, Michie K, Winter AJ, Gunson RN. Hepatitis E in Scotland: assessment of HEV infection in two high-risk patient groups with elevated liver enzymes. J Clin Virol. 2015 Feb;63:36-7.

[38] Payne BA, Medhi M, Ijaz S, Valappil M, Savage EJ, Gill ON, Tedder R, Schwab U. Hepatitis E virus seroprevalence among men who have sex with men, United Kingdom. Emerg Infect Dis. 2013 Feb;19(2):333-5.

[39] Keane F, Gompels M, Bendall R, Drayton R, Jennings L, Black J, Baragwanath G, Lin N, Henley W, Ngui SL, Ijaz S, Dalton H. Hepatitis E virus coinfection in patients with HIV infection. HIV Med. 2012 Jan;13(1):83-8.

[40] Madden RG, Wallace S, Sonderup M, Korsman S, Chivese T, Gavine B, Edem A, Govender R, English N, Kaiyamo C, Lutchman O, van der Eijk AA, Pas SD, Webb GW, Palmer J, Goddard E, Wasserman S, Dalton HR, Spearman CW. Hepatitis E virus: Western Cape, South Africa. World J Gastroenterol. 2016 Nov 28;22(44):9853-9859.

[41] Pineda JA, Cifuentes C, Parra M, Merchante N, Pérez-Navarro E, Rivero-Juárez A, Monje P, Rivero A, Macías J, Real LM. Incidence and natural history of hepatitis E virus coinfection among HIV-infected patients. AIDS. 2014 Aug 24;28(13):1931-7.

[42] Rivero-Juarez A, Martinez-Dueñas L, Martinez-Peinado A, Camacho A, Cifuentes C, Gordon A, Frias M, Torre-Cisneros J, Pineda JA, Rivero A. High hepatitis E virus seroprevalence with absence of chronic infection in HIV-infected patients. J Infect. 2015 Jun;70(6):624-30.

[43] López-Fabal MF, Gómez-Garcés JL. Seroprevalence of hepatitis E virus in patients with hepatitis C and / or infected with HIV. Rev Esp Quimioter. 2015 Dec;28(6):314-6.

[44] Mateos-Lindemann ML, Gonzalez-Galdámez A, Bordallo-Cardona M, Pérez-Gracia MT. Are HIV-infected patients a high-risk population for hepatitis E virus infection in Spain? Enferm Infecc Microbiol Clin. 2012 Nov;30(9):582-3.

[45] Jardi R, Crespo M, Homs M, van den Eynde E, Girones R, Rodriguez-Manzano J, Caballero A, Buti M, Esteban R, Rodriguez-Frias F. HIV, HEV and cirrhosis: evidence of a possible link from eastern Spain. HIV Med. 2012 Jul;13(6):379-83.

[46] Riveiro-Barciela M, Buti M, Homs M, Campos-Varela I, Cantarell C, Crespo M, Castells L, Tabernero D, Quer J, Esteban R, Rodriguez-Frías F. Cirrhosis, liver transplantation and HIV infection are risk factors associated with hepatitis E virus infection. PLoS One. 2014 Jul 28;9(7):e103028.

[47] Mateos-Lindemann ML, Diez-Aguilar M, Galdamez AL, Galán JC, Moreno A, Pérez-Gracia MT. Patients infected with HIV are at high-risk for hepatitis E virus infection in Spain. J Med Virol. 2014 Jan;86(1):71-4.

[48] Kenfak-Foguena A, Schöni-Affolter F, Bürgisser P, Witteck A, Darling KE, Kovari H, Kaiser L, Evison JM, Elzi L, Gurter-De La Fuente V, Jost J, Moradpour D, Abravanel F, Izpopet J, Cavassini M; Data Center of the Swiss HIV Cohort Study, Lausanne, Switzerland. Hepatitis E Virus seroprevalence and chronic infections in patients with HIV, Switzerland. Emerg Infect Dis. 2011 Jun;17(6):1074-8.

[49] Harritshøj LH, Theilgaard ZP, Mannheimer E, Midgley SE, Chiduo M, Ullum H, Katzenstein TL. Hepatitis E virus epidemiology among HIV-infected women in an urban area in Tanzania. Int J Infect Dis. 2018 May 24;73:7-9.

[50] Boon D, Redd AD, Laeyendecker O, Engle RE, Nguyen H, Ocama P, Boaz I, Ndyanabo A, Kiggundu V, Reynolds SJ, Gray RH, Wawer MJ, Purcell RH, Kirk GD, Quinn TC, Stabinski L; Rakai Health Sciences Program. Hepatitis E Virus Seroprevalence and Correlates of Anti-HEV IgG Antibodies in the Rakai District, Uganda. J Infect Dis. 2018 Feb 14;217(5):785-789.

[51] Crum-Cianflone NF, Curry J, Drobeniuc J, Weintrob A, Landrum M, Ganesan A, Bradley W, Agan BK, Kamili S; Infectious Disease Clinical Research Program HIV Working Group. Hepatitis E virus infection in HIV-infected persons. Emerg Infect Dis. 2012 Mar;18(3):502-6.

[52] Sherman KE, Terrault N, Barin B, Rouster SD, Shata MT; HIV-TR Investigators. Hepatitis E infection in HIV-infected liver and kidney transplant candidates. J Viral Hepat. 2014 Aug;21(8):e74-7.

[53] Jacobs C, Chiluba C, Phiri C, Lisulo MM, Chomba M, Hill PC, Ijaz S, Kelly P. Seroepidemiology of hepatitis E virus infection in an urban population in Zambia: strong association with HIV and environmental enteropathy. J Infect Dis. 2014 Mar 1;209(5):652-7.

[54] Lin KY, Lin PH, Sun HY, Chen YT, Su LH, Su YC, et al. (2019) Hepatitis E Virus Infections Among HIV-Positive Individuals During an Outbreak of Acute Hepatitis A in Taiwan. Hepatology. [Epub ahead off print] doi: 10.1002/hep.30771.
